# Analysis of Radiation Dose to the Shoulder by Treatment Technique and Correlation With Patient Reported Outcomes in Patients Receiving Regional Nodal Irradiation

**DOI:** 10.3389/fonc.2021.617926

**Published:** 2021-03-11

**Authors:** Jose G. Bazan, Dominic DiCostanzo, Karen Hock, Sachin Jhawar, Karla Kuhn, Kylee Lindsey, Kayla Tedrick, Erin Healy, Sasha Beyer, Julia R. White

**Affiliations:** ^1^Department of Radiation Oncology, The Ohio State University Comprehensive Cancer Center and Stefanie Spielman Comprehensive Breast Center – Arthur G. James Cancer Hospital and Solove Research Institute, The Ohio State University, Columbus, OH, United States; ^2^Department of Physical Therapy, The Ohio State University Comprehensive Cancer Center and Stefanie Spielman Comprehensive Breast Center – Arthur G. James Cancer Hospital and Solove Research Institute, The Ohio State University, Columbus, OH, United States

**Keywords:** IMRT, shoulder, 3DCRT, quick DASH, PMRT, RNI

## Abstract

**Background/Purpose:**

Shoulder/arm morbidity is a late complication of breast cancer treatment with surgery and regional nodal irradiation (RNI). We set to analyze the impact of radiation technique [intensity modulated radiation therapy (IMRT) or 3D conformal radiation therapy (3DCRT)] on radiation dose to the shoulder with a hypothesis that IMRT use results in smaller volume of shoulder receiving radiation. We explored the relationship of treatment technique on long-term patient-reported outcomes using the quick disabilities of the arm, shoulder, and hand (q-DASH) questionnaire.

**Materials/Methods:**

We identified patients treated with adjuvant RNI (50 Gy/25 fractions) from 2013 to 2018. We retrospectively contoured the shoulder organ-at-risk (OAR) from 2 cm above the ipsilateral supraclavicular (SCL) planning target volume (PTV) to the inferior SCL PTV slice and calculated the absolute volume of shoulder OAR receiving 5–50 Gy (V5–V50). We identified patients that completed a q-DASH questionnaire ≥6 months from the end of RNI.

**Results:**

We included 410 RNI patients: 54% stage III, 72% mastectomy, 35% treated with IMRT. IMRT resulted in significant reductions in the shoulder OAR volume receiving 20–50 Gy *vs.* 3DCRT. In total, 82 patients completed the q-DASH. The mean (SD) q-DASH=25.4 (19.1) and tended to be lower with IMRT *vs.* 3DCRT: 19.6 (16.4) *vs.* 27.8 (19.8), p=0.078.

**Conclusion:**

We found that IMRT reduces radiation dose to the shoulder and is associated with a trend toward reduced q-DASH scores ≥6 months post-RNI in a subset of our cohort. These results support prospective evaluation of IMRT as a technique to reduce shoulder morbidity in breast cancer patients receiving RNI.

## Introduction

Regional nodal irradiation (RNI) in breast cancer patients is expanding based on clinical trials and meta-analyses (1–3). Shoulder and arm morbidity is an important late effect of whole breast irradiation and RNI (4–12). Shoulder and arm morbidity include impairments of shoulder/arm movement, chest wall discomfort, lymphedema, and upper extremity weakness (6) which negatively impact quality of life (9, 13). These morbidities are common with as many as 2/3 of patients reporting decreased shoulder mobility and nearly 20% of patients experiencing persistent shoulder/arm pain more than 2.5 years out from the end of radiation (10).

Muscles with origins or insertions on the humerus constitute a complex system—any one of these muscles that gets exposed to radiation can affect shoulder function. Compared to whole breast or chestwall-only radiation, RNI increases dose to the muscles of the shoulder and upper back (14). Intensity modulated radiation therapy (IMRT) is a planning technique that conforms dose to targets and limits high/moderate radiation doses to adjacent organs-at-risk (OARs) and is sometimes used for RNI in order to meet heart or lung constraints (15, 16). By default, IMRT may reduce the volume of shoulder musculature exposed to high/moderate radiation doses relative to 3D conformal radiation therapy (3DCRT), but this has not been previously characterized.

While it is clear that RNI increases dose to the shoulder, the relationship between the volume of shoulder exposed to various radiation doses with shoulder morbidity is not well characterized. Evaluation of shoulder function with a validated tool is lacking in breast cancer patients receiving RNI. The disabilities of the arm, shoulder, and hand (DASH) questionnaire is a 30-item evaluation tool to measure arm/shoulder/hand morbidity with lower scores (100-point scale) indicating less morbidity. Questions assess ability to complete common tasks (e.g. opening a jar) as well as the presence of pain and/or weakness. A systematic review of the literature recommends use of the DASH to measure shoulder/arm dysfunction in breast cancer patients (17). The quick-DASH (q-DASH) is a shorter (11-item) tool that results in similar scores to the DASH and is increasingly being used in the clinic (18, 19).

Here, we aimed to study the impact of radiation treatment technique (IMRT *vs.* 3DCRT) on radiation dose to the shoulder with a hypothesis that IMRT results in lower volume of the shoulder receiving high/moderate doses of radiation. We further set to characterize long-term (≥6 months) shoulder morbidity in patients that had completed the q-DASH. Lastly, we compare q-DASH scores in patients treated with 3DCRT *versus* IMRT to test the hypothesis that the expected reduction in the volume of shoulder receiving high/moderate doses of radiation with IMRT would translate to improved (lower) q-DASH scores.

## Materials and Methods

### Patient Selection

We obtained Institutional Review Board approval for this study (IRB #2018C0011). We used our departmental database to identify all patients treated with RNI with conventional fractionation (200 cGy/fraction) from 1/2013 to 12/2018. Patient were excluded for the following: recurrent disease; simultaneous distant metastatic disease; hypofractionated radiation; re-irradiation regimens, boost to extra-axillary disease in the supraclavicular fossa and/or axillary apex; bilateral RNI.

### Radiation Simulation and Treatment Planning

Details of our RNI treatment planning algorithm have been described previously (20, 21). Briefly, patients underwent CT simulation in the supine position (or prone in select cases) with a free-breathing CT scan (FBCT). An additional deep inspiration breath hold (DIBH) scan was obtained for all left-sided and select right-sided cases. The target volumes were contoured per the NSABP B51/RTOG 1304 clinical trial guidelines, which is based on the RTOG Contouring Atlas (22, 23). The OARs prospectively and routinely contoured were: whole heart; bilateral lungs; contralateral breast/chestwall, and thyroid. There was no prospectively contoured OAR to account for muscles/soft tissues of the neck, shoulder, or back. The prescription dose was 5,000 cGy in 25 fractions to the breast/chestwall and regional nodes. An additional boost to the mastectomy scar or lumpectomy cavity was given per treatment physician discretion. We followed the planning objectives and normal tissue constraints of NSABP B51/RTOG 1304 trial for all plan evaluation and approval. The treatment planning algorithm begins with 3DCRT but changes to IMRT as needed to meet critical heart/lung planning constraints.

### Definition of the Shoulder OAR and Back OARs

**Figure 1:**
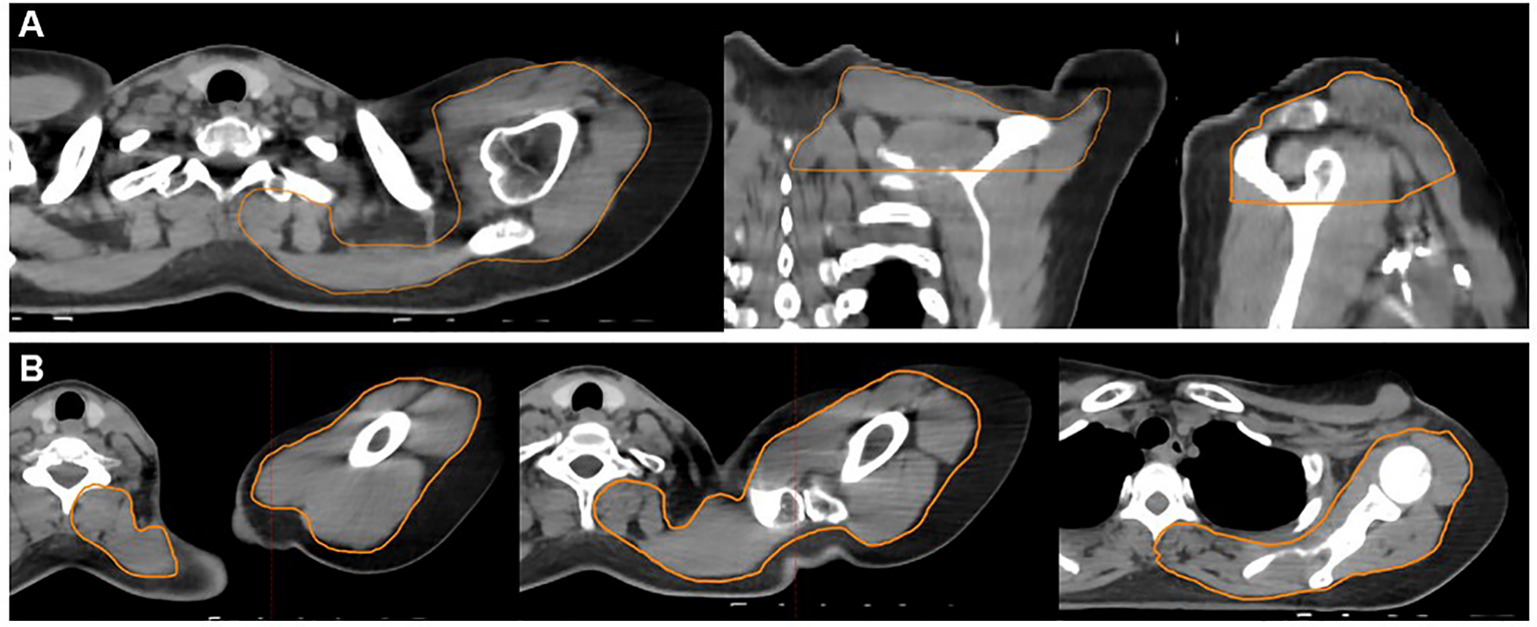
Example contours of the shoulder organ-at-risk (OAR) (orange contour). The shoulder OAR structure begins 2 cm above the cranial slice of the supraclavicular PTV ends at the inferior slice of the supraclavicular PTV. Panel **(A)** demonstrates an axial (left), coronal (middle), and sagittal (right) projections. Panel **(B)** demonstrates axial slices only progressing from the superior (far left) to inferior (far right) extent of the shoulder OAR. These structures include all muscle, soft tissue, bony and vascular structures of the shoulder and back, excluding the spine and ribs.

**Figure 2:**
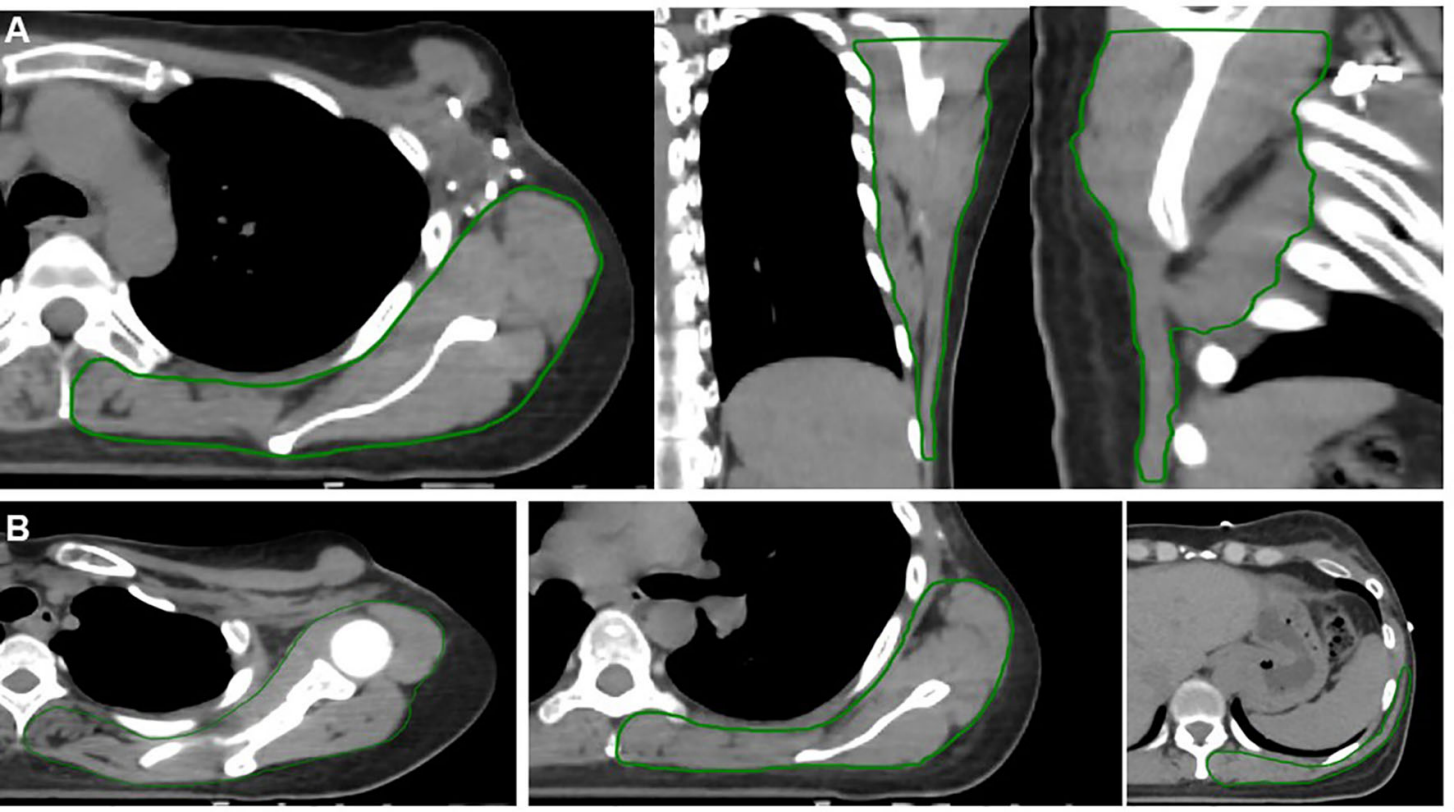
Example contours of the back organ-at-risk (OAR) (green contour). The back OAR extends 2 cm inferiorly from the most caudal slice of the chestwall/breast PTV. Panel **(A)** demonstrates an axial (left), coronal (middle), and sagittal (right) projections. Panel **(B)** demonstrates axial slices only progressing from the superior (far left) to inferior (far right) extent of the back OAR. These structures include all muscle, soft tissue, bony and vascular structures of the shoulder and back, excluding the spine and ribs.

For each case, we retrospectively contoured the muscles and soft tissues of the neck, shoulder, and back. Few RNI studies have characterized the shoulder as an OAR, and therefore, there is no consensus definition for the shoulder OAR. Johansen et al. defined a shoulder OAR by contouring the outer border of the humerus, the coracoid process, and the acromion with a 5 mm margin (24). In a separate study by Lipps et al., nine individual muscles of the shoulder and chestwall were contoured as separate structures (14). In our study, we decided that the shoulder OAR and back OAR structures should include muscles, soft tissues, bone, and vasculature since all of these are integral components to the structure and function of the shoulder, and all can be affected by radiation. We defined the shoulder OAR as all muscle, soft tissues, vasculature, and bones (excluding the vertebra) in the posterior neck, shoulder, and upper arm region beginning from 2 cm superior to the most cranial slice of the supraclavicular (SCL) PTV and ending at the most caudal SCL PTV slice (**Figures 1A, B**). Partial or whole muscles included in the shoulder OAR included: trapezius, levator scapulae, deep cervical muscles, posterior scalene, biceps brachii, deltoid, subscapularis, infraspinatus, latissimus dorsi, pectoralis major. In addition, we defined the back OAR as all posterior chestwall muscle, soft tissue, vasculature and bones (excluding the ribs and vertebra) beginning one slice inferior to the caudal slice of the SCL PTV and extending to 2 cm inferior to the most caudal chestwall/PTV slice (**Figures 2A, B**). The anterior most extent of the posterior chestwall OAR was the anterior edge of the latissimus dorsi muscle (pectoralis muscles and the other intercostal muscles anterior to the latissimus were not included). Partial or whole muscles included in the back OAR included the: latissimus dorsi, serratus anterior, subscapularis, infraspinatus, supraspinatus, trapezius, erector spinae. Portions of certain muscles could be included in both the shoulder OAR and back OAR due to their length and size (e.g., latissimus dorsi, trapezius, subscapularis). Last, we defined the shoulder+back OAR as the union of the shoulder OAR and back OAR.

### Data Collection, Outcomes, and Statistics

**Figure 3:**
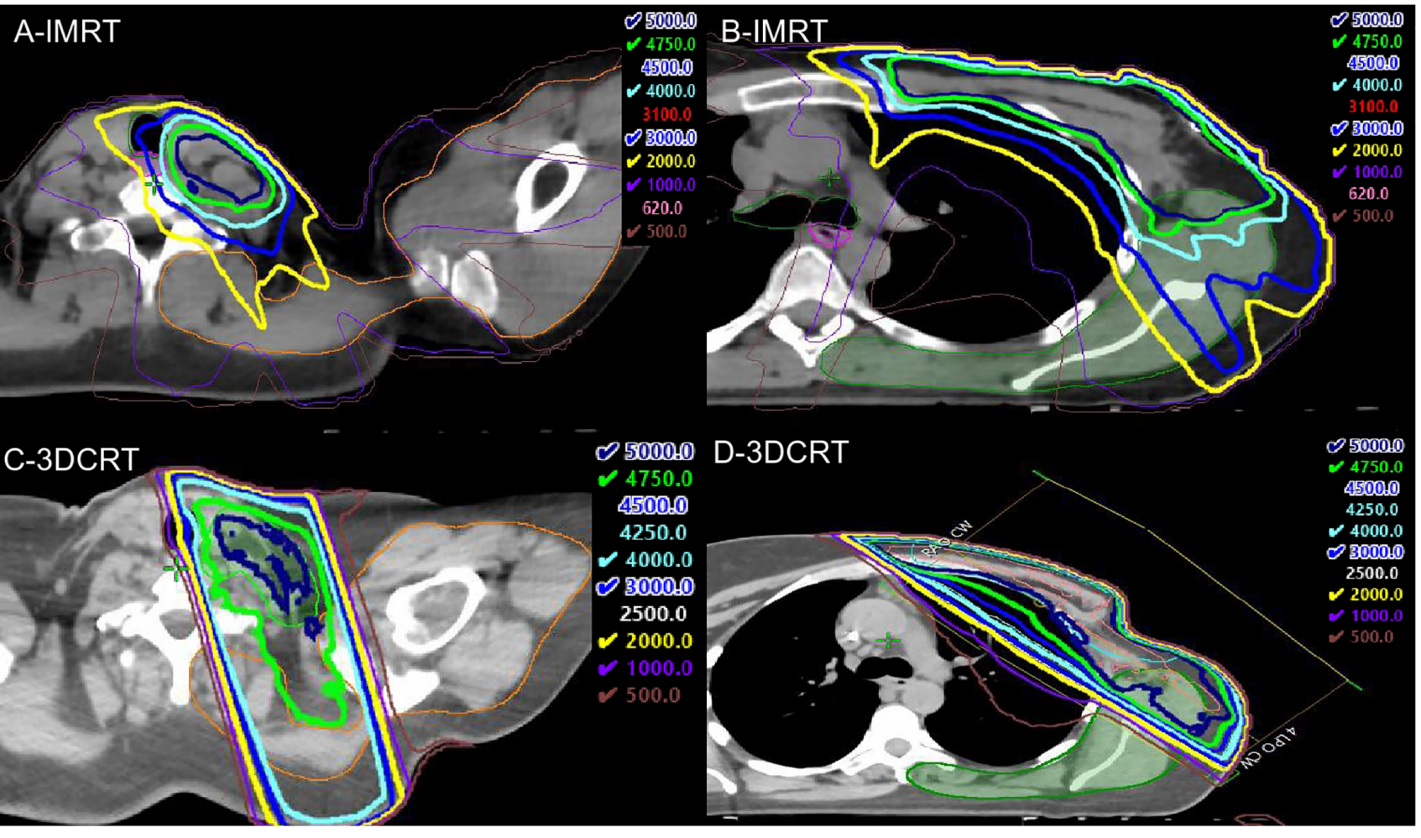
Representative intensity modulated radiation therapy (IMRT) (top panels) and 3DCRT (bottom panels) dose distributions. **(A)** shows that the 30Gy (light blue), 40 Gy (cyan), 47.5 Gy (green), and 50Gy (dark blue) isodose lines nearly exclude the shoulder OAR (orange contour), and a small portion of the shoulder OAR receives 20 Gy (yellow). In contrast, the 20–47.5 Gy isodose lines extend far posteriorly to include the shoulder OAR with 3DCRT **(C)**. In **(B)**, the 40–50 Gy isodose lines exclude the majority of the back OAR (green contour), but there are projections of the 30 and 20 Gy isodose lines that cover a significant portion of the back OAR compared to the 3DCRT patient **(D)**.

First, we aimed to compare the volume of shoulder OAR and back OAR receiving various radiation doses between IMRT and 3DCRT patients. The dose volume histogram (DVH) of the final plan was then analyzed to capture the absolute volume of shoulder OAR, back OAR and back+shoulder OAR (in cc) receiving at least 5 Gy, 10 Gy, 20 Gy, 30 Gy, 40 Gy, 47.5 Gy, and 50 Gy (V5–V50). **Figure 3** shows a typical dose distribution for IMRT (**Figures 3A, B**) and for 3DCRT (**Figures 3C, D**). Due to the standard 3DCRT beam arrangement of anterior and posterior oblique fields to treat the SCL and axillary apical nodes (**Figure 3**), we hypothesized that IMRT patients would have a lower volume of shoulder OAR exposed to moderate-high dose radiation (V20-V50) compared to 3DCRT patients. Since tangential fields are used to treat the breast/chestwall (**Figure 3**), we hypothesized that the volume of back OAR exposed to moderate-high dose radiation would be similar between IMRT and 3DCRT patients. Differences in volume between IMRT and 3DCRT patients were analyzed with a t-test (p<0.05 considered statistically significant).

Next, we aimed to describe the late (≥6 month) shoulder/arm morbidity in patients using a patient-reported outcome measure. We reviewed each eligible patient’s electronic medical record (EMR) to determine whether the patient had completed a q-DASH questionnaire during their disease course. The q-DASH is performed only in patients that had documented visits with physical therapy (PT) in our institution and is entered into the EMR. The q-DASH contains 11 items, scored on a 0–5 scale, and at least 10 of the 11 items must be completed for a score to be calculated. The scores are summed and averaged and the converted to a 100-point scale by subtracting 1 from the average and multiplying by 25:

(25) [(sum of  n   reponses/n) – 1] × 25

The time point at which PT assessments were done were collected for each patient and categorized as: pre-surgery, post-surgery/pre-radiation, and/or post-radiation. Pre-surgery PT assessments were routine to establish baseline measurements. Post-surgery/pre-radiation and post-radiation PT assessments were not routine and were based on referrals for lymphedema and/or decreased range of motion. We recorded the q-DASH if it had been completed ≥6 months from the end of RNI. Differences in patient characteristics between those that completed the q-DASH and those that did not were compared using a t-test (continuous variables) or chi-square test (categorical variables). Summary statistics were used to describe the q-DASH scores. We used a t-test to compare the q-DASH in patients treated with 3DCRT *versus* IMRT. All statistical analyses were performed using SAS version 9.4 (Carey, NC).

## Results

### Patient Characteristics

From 1/2013 to 12/2018, 459 patients received RNI. Of these, 49 were excluded: 20 (recurrent disease); 17 (SCL or axillary nodal boost); 5 (hypofractionated radiation or re-irradiation); 4 (bilateral RNI); 3 (simultaneous distant metastases). The remaining 410 patients met the study criteria with characteristics summarized in **Table 1**. The majority of patients had stage III disease (54%) and underwent mastectomy (72%). Most patients (83%) had an axillary lymph node dissection with a mean of 18.7 nodes (SD=11.2) removed. Overall, 90% of patients received chemotherapy and nearly all ER+ or PR+ patients received endocrine therapy (99%). In terms of treatment technique, 35% (N=145) of patients were treated with IMRT and the remaining 65% (N=265) were treated with 3DCRT. **Table 1** demonstrates that patients treated with IMRT had a significantly higher mean age (55.1 *vs.* 52.1 years, p=0.01), were more likely to have left sided disease (57 *vs.* 46%, p=0.02), and were more likely to receive a mastectomy scar boost (57% *vs.* 42%, p=0.01) compared to patients treated with 3DCRT. The rest of the baseline characteristics were similar between the two groups.

**Table 1:** Patient characteristics by treatment technique (intensity modulated radiation therapy *versus* 3D conformal radiation therapy).

	Entire cohort (N = 410)	IMRT (N = 145)	3DCRT (N = 265)	p-value
**Age, mean (SD)**	53.1 years (11.1)	55.1 (11.4)	52.0 (10.8)	0.01
**Stage, no. (%)** II III	187 (46%) 223 (54%)	61 (42%) 84 (58%)	126 (48%) 139 (52%)	0.29
**Subtype** ER+ or PR +/HER2− Triple negative HER2+	247 (60%) 74 (18%) 89 (22%)	85 (59%) 31 (21%) 29 (20%)	162 (61%) 43 (16%) 60 (23%)	0.41
**Laterality** Left Right	204 (50%) 206 (50%)	83 (57%) 62 (43%)	121 (46%) 144 (54%)	0.02
**Grade** 1–2 3	223 (54%) 187 (46%)	72 (50%) 73 (50%)	151 (57%) 114 (43%)	0.15
**Histology** IDC ILC/mixed	353 (86%) 57 (14%)	123 (85%) 22 (15%)	230 (87%) 35 (13%)	0.73
**Type of surgery** Mastectomy Lumpectomy	297 (72%) 113 (28%)	110 (76%) 35 (24%)	187 (71%) 78 (29%)	0.25
**Axillary surgery** ALND SLNBx	341 (83%) 69 (17%)	125 (86%) 20 (14%)	216 (82%) 49 (18%)	0.22
**Nodes removed** Mean (SD)	18.7 (11.2)	19.2 (10.5)	18.4 (11.5)	0.46
**Chemotherapy** Preoperative Postoperative No chemotherapy	200 (49%) 168 (41%) 42 (10%)	73 (50%) 60 (41%) 12 (9%)	127 (48%) 108 (41%) 30 (11%)	0.62
**Endocrine therapy** Yes No Not applicable	309 (73%) 4 (1%) 97 (24%)	106 (73%) 1 (1%) 38 (26%)	203 (77%) 3 (1%) 59 (22%)	0.62
**Radiation boost** Lump cavity (n=113) Yes No Mast scar (n=297) Yes No	104 (92%) 9 (8%) 145 (49%) 152 (51%)	34 (97%) 1 (3%) 64 (58%) 46 (42%)	70 (90%) 8 (10%) 81 (43%) 106 (57%)	0.18 0.01
**PT assessments** Pre-surgery Pre-RT Post-RT All three time points No assessments	195 (48%) 284 (69%) 241 (59%) 104 (25%) 50 (12%)	67 (46%) 109 (75%) 84 (58%) 36 (25%) 12 (8%)	128 (48%) 175 (66%) 157 (59%) 68 (26%) 38 (14%)	0.68 0.05 0.80 0.85 0.07

SD, standard deviation; IDC, infiltrating ductal carcinoma; ILC, invasive lobular carcinoma; ALND, axillary lymph node dissection; SLNBx, sentinel lymph node biopsy; 3DCRT, 3D conformal radiation therapy; IMRT, intensity modulated radiation therapy; PT, physical therapy.

### Radiation Dose to the Shoulder and Back

**Table 2** summarizes the volume of shoulder OAR, back OAR, and shoulder+back OAR that received 5–50 Gy in the IMRT and 3DCRT patients. IMRT resulted in a significantly reduced volume of shoulder OAR exposed to moderate and high doses of radiation therapy (V20–V50 Gy). IMRT also resulted in a lower volume of back OAR receiving 40–50 Gy and a non-significant decrease in back OAR volume receiving 30 Gy. The V5–10 of the shoulder and back OAR were significantly higher with IMRT and the V20 of the back OAR was also higher with IMRT. When examining the combined shoulder+back OAR, V20–V50 were all significantly lower in IMRT patients.

**Table 2:** Absolute volume of shoulder, back, and shoulder+back receiving radiation by treatment technique.

Dose parameter	IMRT (n = 145)	3DCRT (n = 265)	p-value
**V50Gy, mean (SD) cc** Shoulder+back OAR Shoulder OAR Back OAR	38.6 (35.1) 12.1 (17.2) 26.1 (27.3)	120.3 (88.6) 40.2 (52.6) 79.5 (56.1)	<0.0001 <0.0001 <0.0001
**V47.5Gy, mean (SD) cc** Shoulder+back OAR Shoulder OAR Back OAR	64.1 (54.5) 23.8 (32.5) 39.4 (35.8)	269.6 (142.1) 149.4 (96.1) 117.7 (74.2)	<0.0001 <0.0001 <0.0001
**V40Gy, mean (SD) cc** Shoulder+back OAR Shoulder OAR Back OAR	132.7 (88.9) 53.9 (54.0) 77.4 (55.8)	458.6 (140.7) 289.7 (91.4) 164.2 (88.3)	<0.0001 <0.0001 <0.0001
**V30Gy, mean (SD) cc** Shoulder+back OAR Shoulder OAR Back OAR	301.5 (138.9) 133.5 (90.9) 172.0 (110.9)	526.6 (149.8) 330.8 (98.4) 190.2 (93.3)	<0.0001 <0.0001 0.0777
**V20Gy, mean (SD) cc** Shoulder+back OAR Shoulder OAR Back OAR	570.5 (185.3) 282.3 (121.4) 284.2 (120.9)	611.7 (170.9) 391.9 (172.6) 222.3 (102.8)	.0240 <0.0001 <0.0001
**V10Gy, mean (SD) cc** Shoulder+back OAR Shoulder OAR Back OAR	952.3 (238.0) 489.7 (136.0) 459.0 (160.4)	689.0 (191.4) 421.9 (120.8) 260.7 (115.0)	<0.0001 <0.0001 <0.0001
**V5Gy, mean (SD) cc** Shoulder+back OAR Shoulder OAR Back OAR	1224.8 (269.9) 606.9 (130.1) 611.3 (189.7)	789.0 (220.3) 467.9 (130.4) 314.5 (134.5)	<0.0001 <0.0001 <0.0001

Vx, volume of muscle receiving x Gy or higher; IMRT, intensity-modulated radiation therapy; 3DCRT, 3D conformal radiation therapy; SD, standard deviation.

In order to take into account the fact that the volume of the shoulder OAR and/or back OAR may vary from patient to patient based on individual anatomy and on the superior/inferior extent of the SCL PTV, we also examined the relative volume of the shoulder OAR, back OAR, and shoulder+back OAR receiving 5–50 Gy. **Table 3** shows that the relative volume of shoulder OAR receiving 20–50 Gy was significantly lower in patients that received IMRT. For example, the mean (standard deviation) V30 was 15.3% (9.5%) in patients treated with IMRT compared to 37.1% (8.1%) in patients treated with 3DCRT. Similar to the results seen when measured in absolute volume, the relative V40–V50 of the back OAR were lower in patients treated with IMRT compared to 3DCRT. The relative V5–V10 of all structures were significantly higher in patients that received IMRT compared to 3DCRT.

**Table 3:** Relative volume of shoulder, back and shoulder+back receiving radiation by treatment technique.

Dose parameter	IMRT (n = 145)	3DCRT (n = 265)	p-value
**V50Gy (%), mean (SD)** Shoulder+back OAR Shoulder OAR Back OAR	2.2 (2.0) 1.3 (1.6) 3.0 (3.0)	6.5 (4.2) 4.4 (5.4) 8.6 (5.2)	<0.0001 <0.0001 <0.0001
**V47.5Gy, mean (SD) cc** Shoulder+back OAR Shoulder OAR Back OAR	3.7 (3.2) 2.7 (3.1) 4.6 (4.0)	14.6 (6.2) 16.4 (9.5) 12.6 (6.2)	<0.0001 <0.0001 <0.0001
**V40Gy, mean (SD) cc** Shoulder+back OAR Shoulder OAR Back OAR	7.7 (4.8) 6.2 (5.8) 8.9 (6.1)	25.4 (4.9) 32.4 (7.7) 17.8 (7.0)	<0.0001 <0.0001 <0.0001
**V30Gy, mean (SD) cc** Shoulder+back OAR Shoulder OAR Back OAR	17.3 (6.9) 15.3 (9.5) 19.6 (11.0)	29.2 (5.0) 37.1 (8.1) 20.7 (7.2)	<0.0001 <0.0001 0.2268
**V20Gy, mean (SD) cc** Shoulder+back OAR Shoulder OAR Back OAR	32.7 (7.9) 32.4 (11.5) 32.5 (11.7)	33.9 (5.4) 43.0 (8.9) 24.2 (7.6)	.0722 <0.0001 <0.0001
**V10Gy, mean (SD) cc** Shoulder+back OAR Shoulder OAR Back OAR	54.8 (8.3) 56.6 (10.1) 52.6 (13.2)	38.2 (5.9) 47.3 (9.4) 28.4 (8.2)	<0.0001 <0.0001 <0.0001
**V5Gy, mean (SD) cc** Shoulder+back OAR Shoulder OAR Back OAR	70.7 (7.8) 70.7 (8.6) 70.0 (12.4)	43.7 (6.7) 52.5 (10.0) 34.3 (9.2)	<0.0001 <0.0001 <0.0001

Vx, volume of muscle receiving x Gy or higher; IMRT, intensity-modulated radiation therapy; 3DCRT, 3D conformal radiation therapy; SD, standard deviation.

### Objective Measures of Shoulder/Arm Function And Patient Reported Outcomes Using the Quick DASH

PT evaluations were performed in about 50% of patients prior to surgery, 70% post-surgery prior to the start of RNI, and 60% post-RNI (**Table 1**). Only 25% of patients had a PT assessment at all three time points and 12% of patients had no PT assessment at any time point. Patients treated with IMRT were more likely to have a pre-radiation PT assessment compared to patients treated with 3DCRT (75 *vs.* 66%, p=0.05), but there was no significant difference in the rate of post-radiation PT assessments by treatment technique (58% IMRT *vs.* 59% 3DCRT, p=0.80).

The incidence of post-RNI lymphedema and decreased range of motion, as confirmed by physical therapy evaluation, were 36.8 and 33.4%, respectively. There was no significant difference in lymphedema by type of RNI (42.1% IMRT *vs.* 34.0% 3DCRT, p=0.10). Similarly, the rates of decreased range of motion were similar by type of RNI (34.5% IMRT *vs.* 32.8% 3DCRT, p=0.73).

Of the 410 patients, 82 (20%) completed the q-DASH≥6 months post-RNI as part of an evaluation in PT. These 82 patients were referred to PT post-treatment for lymphedema (59%), decreased range of motion (27%), both lymphedema and range of motion problems (8%), or other reasons (6%). The median time of q-DASH completion was 13 months (IQR, 9–22 months) post-RNI. Overall, 39 patients (48%) completed the q-DASH from 6 months to 1 year post-RNI, 28 patients (34%) completed it between 1 and 2 years post-RNI, and the remaining 15 patients (18%) completed it >2 years post-RNI. **Table 4** demonstrates that patients with a q-DASH were more likely to have undergone mastectomy (82 *vs.* 70%, p=0.04) and had a trend toward higher rates of ALND (90 *vs.* 81%, p=0.06) compared to those that did not have a q-DASH. In addition, q-DASH patients were more likely to have received a PT assessment at each time point compared to patients without a q-DASH (**Table 4**). Breast cancer subtype was unevenly distributed between the groups with fewer (ER+ or PR+)/HER2− patients in the q-DASH group (p=0.01). However, the median number of axillary nodes removed was similar between the groups and there were no other differences in patient or treatment characteristics that could impact shoulder morbidity (age, use of systemic therapy, radiation technique, radiation boost).

**Table 4:** Patient characteristics in patients that completed the quick disabilities of the arm, shoulder, and hand (DASH) questionnaire compared to those patients that did not complete a quick-DASH.

	Quick DASH (N = 82)	No quick DASH (N = 328)	p-value
**Age, mean (SD)**	53.0 years (10.2)	53.1 years (11.4)	0.90
**Stage, no. (%)** II III	32 (39%) 50 (61%)	155 (47%) 173 (53%)	0.94
**Subtype** ER+ or PR+/HER2− Triple negative HER2+	44 (54%) 10 (12%) 28 (34%)	203 (62%) 64 (20%) 61 (18%)	0.01
**Laterality** Left Right	40 (49%) 42 (51%)	164 (50%) 164 (50%)	0.84
**Grade** 1–2 3	42 (51%) 40 (49%)	181 (55%) 147 (45%)	0.52
**Histology** IDC ILC/mixed	73 (89%) 9 (11%)	280 (85%) 48 (15%)	0.39
**Type of surgery** Mastectomy Lumpectomy	67 (82%) 15 (18%)	230 (70%) 98 (30%)	0.04
**Axillary surgery** ALND SLNBx	74 (90%) 8 (10%)	267 (81%) 61 (19%)	0.06
**Nodes removed** Mean (SD)	20.4 (10.7)	18.2 (11.2)	0.12
**Chemotherapy** Preoperative Postoperative No chemotherapy	41 (50%) 35 (43%) 6 (7%)	159 (48%) 133 (41%) 36 (11%)	0.62
**Endocrine therapy** Yes No Not applicable	60 (73%) 1 (1%) 21 (26%)	249 (76%) 3 (1%) 76 (23%)	0.86
**RT technique** 3DCRT IMRT	58 (71%) 24 (29%)	207 (63%) 121 (37%)	0.20
**Radiation boost** Lump cavity (n=113) Yes No Mast scar (n=297) Yes No	14 (93%) 1 (7%) 30 (45%) 37 (55%)	90 (92%) 8 (8%) 115 (50%) 115 (50%)	0.84 0.45
**PT assessments** Pre-surgery Pre-RT Post-RT All three time points No assessments	47 (57%) 66 (80%) 82 (100%) 42 (51%) 0 (1%)	148 (45%) 218 (66%) 159 (48%) 62 (19%) 50 (15%)	0.048 0.014 <0.0001 <0.0001 0.0002

SD, standard deviation; IDC, infiltrating ductal carcinoma; ILC, invasive lobular carcinoma; ALND, axillary lymph node dissection; SLNBx, sentinel lymph node biopsy; 3DCRT, 3D conformal radiation therapy; IMRT, intensity modulated radiation therapy; PT, physical therapy.

Amongst the 82 patients that completed the q-DASH, the mean score (SD) was 25.4 (19.1) and median (IQR) was 20.7 (9.1–38.6). The q-DASH scores in patients treated with mastectomy (N=67) were mean 25.4 (18.6) and median 20.8 (9.1–38.6) while the mean score was 25.5 (22.2) and median 20.5 (4.5–47.7) in the 15 patients treated with lumpectomy. There were 58 patients treated with 3DCRT and 24 patients treated with IMRT. Similar to the entire cohort of patients, the relative or absolute shoulder V20–V50 were significantly lower in patients treated with IMRT compared to those that received 3DCRT (**Table 5**). The mean q-DASH scores tended to be lower in patients treated with IMRT compared to 3DCRT at 19.6 (16.4) *vs.* 27.8 (19.8), p=0.078.

**Table 5:** Volume of shoulder organ-at-risk (OAR) receiving moderate to high-dose radiation by type of radiation therapy in patients with quick disabilities of the arm, shoulder, and hand (DASH) scores.

Dose parameter	IMRT (n = 24)	3DCRT (n = 58)	p-value
V50Gy (SD), cc V50Gy (SD), %	8.8 (10.6) 1.0% (1.0)	35.7 (45.5) 4.0% (4.9)	0.0056 <0.0001
V47.5Gy (SD), cc V47.5Gy (SD), %	16.7 (17.3) 1.9% (1.5)	140.5 (93.5) 15.5% (9.3)	<0.0001 <0.0001
V40Gy (SD), cc V40Gy (SD), %	41.9 (36.4) 4.7% (3.2)	282.7 (82.9) 32.0% (7.0)	<0.0001 <0.0001
V30Gy (SD), cc V30Gy (SD), %	104.0 (66.0) 11.9% (6.0)	325.9 (90.0) 37.0% (7.5)	<0.0001 <0.0001
V20Gy (SD), cc V20Gy (SD), %	223.9 (96.9) 25.6% (7.9)	377.5 (103.0) 42.8% (8.3)	<0.0001 <0.0001
V10Gy (SD), cc V10Gy (SD), %	458.0 (150.6) 52.7% (8.7)	414.6 (111.3) 47.1% (8.9)	0.1531 0.0099
V5Gy (SD), cc V5Gy (SD), %	578.3 (135.3) 67.7 (6.3)	460.6 (121.7) 52.2 (9.6)	0.0002 <0.0001

Vx, volume of muscle receiving x Gy or higher; IMRT, intensity-modulated radiation therapy; 3DCRT, 3D conformal radiation therapy; SD, standard deviation.

Last, we performed an additional exploratory analysis in the 24 patients that had both pre-radiation and post-radiation q-DASH assessments. Of these 24 patients, 13 were treated with 3DCRT and 11 with IMRT. The q-DASH score increased in 85% of the 3DCRT patients compared to 64% of the IMRT patients (p=0.24). The mean change in the q-DASH was +2.2 (SD +25) in IMRT patients and +12.6 (SD +14.4) in the 3DCRT patients (p=0.21).

## Discussion

In this study, we found that IMRT significantly reduces the volume of shoulder OAR receiving moderate and high dose radiation (V20–V50 Gy) compared to patients treated with 3DCRT, consistent with our primary hypothesis. This reduction in volume of shoulder exposed to moderate/high-dose radiation occurred without a specific planning objective placed on the shoulder OAR structure during IMRT optimization. Next, we found that in patients that had a q-DASH score obtained at least 6 months from the end of radiation, shoulder morbidity as measured by the q-DASH tended to be lower in the patients that received IMRT compared to those that received 3DCRT, although this was not statistically significant. To our knowledge, this is the first study to comprehensively evaluate radiation dose to the shoulder and back by treatment technique (IMRT *vs.* 3DCRT) and to examine the impact of treatment technique on patient-reported shoulder morbidity.

The relationship between radiation dose to the shoulder and morbidity is poorly understood. Johansen et al. used the Kwan’s Arm Problem Scale (KAPS) to assess arm/shoulder morbidity and found that the volume of shoulder receiving 15 Gy was associated with higher (worse) KAPS score and a shoulder abduction difference of ≥25 degrees (24). Lipps et al. performed robot-assisted biomechanical measures of shoulder stiffness in nine patients that received RNI using 3DCRT and found no significant difference in shoulder stiffness when compared to 9 patients that received whole breast-only 3DCRT or nine health controls (26). However, there was a significant correlation between radiation dose ≥40 Gy to the pectoralis major muscle and increased stiffness of the muscle (measured by ultrasound shockwave elastography) in both the sternocostal and clavicular fiber regions. While the authors reason that RNI patients develop compensatory mechanisms in other shoulder muscles to stabilize the shoulder joint, the study may have been too small to detect significant differences in the specific biomechanical assessments of the shoulder joint. No other studies have evaluated radiation dose to the shoulder and correlated with functional and/or patient-reported outcomes.

In addition to lack of a uniform shoulder OAR definition, progress in understanding and reducing RNI-related shoulder morbidity has been delayed due to the lack of a consistent measure to assess shoulder function. With respect to objective measures of shoulder and arm morbidity, we found that a relatively high proportion (>30%) of our patient population were treated by physical therapy for lymphedema and/or decreased range of motion of the ipsilateral shoulder post-RNI. There was no difference in rates of these morbidities by treatment technique. One of the weaknesses of using these objective measures is that we cannot adequately capture the severity of the impaired range of motion or lymphedema because these morbidities were not graded according to standard tools used in oncology such as the Common Terminology Criteria for Adverse Events (CTCAE). However, the CTCAE grades lymphedema without respect to absolute or relative changes in limb volume and in such a way that it would still be difficult to quantify subtle differences in severity between patients (grade 1: trace thickening or faint discoloration; grade 2: marked discoloration OR leathery skin texture OR papillary formation OR limiting instrumental activities of daily living; grade 3: severe symptoms OR limiting self-care activities of daily living). Similarly, the CTCAE criteria for joint range of motion (grade 1: ≤25% loss of ROM; grade 2: >25–50% decrease in ROM OR limiting instrumental ADL; grade 3: >50% decrease in ROM OR limiting self-care ADL) does not effectively capture the complexity of all the movements in the shoulder joint such and potentially misses the impact on patient quality of life and function.

As an alternative to purely objective measures, large, randomized trials have also used different patient-reported outcomes tools to assess shoulder morbidity (13, 27). Therefore, in addition to quantifying radiation dose to the shoulder OAR in this study, we also evaluated shoulder morbidity in patients using a simplified version of the DASH (quick DASH), a tool that is now recommended to be used routinely to assess shoulder morbidity in breast cancer patients (17). Prior studies have shown that DASH scores may be has high as 19 after mastectomy with adjuvant RT (5) or as low as 10 in mastectomy without RT (28), and there are no studies of DASH scores in women that have undergone lumpectomy+RNI. Our study is therefore the first to characterize these scores in RNI patients and by treatment technique. We found that RNI patients had a mean q-DASH score>25 at least 6 months post-radiation, indicating significant shoulder morbidity. However, these scores should be interpreted with caution as all of these scores were obtained in patients that had been referred to PT post-RNI with 94% experiencing lymphedema and/or arm/shoulder problems. As such, we would expect that these q-DASH scores are higher (indicating more morbidity) than those of the rest of our study population. Despite this, we felt it worthwhile to report the q-DASH scores since there are few existing data in the literature. In addition, even in patients experiencing symptoms that prompted PT referrals, we saw a trend toward lower scores in the patients treated with IMRT compared to those treated with 3DCRT. In the smaller subset of 24 patients that had pre-radiation and post-radiation q-DASH scores, we also saw numerically smaller changes in q-DASH scores and a smaller proportion of patients that had worsening of q-DASH scores with IMRT compared to 3DCRT. These data remain hypothesis generating and need to be measured consistently and prospectively pre-radiation and post-radiation in order to determine if IMRT use can lessen the impact of RNI on shoulder morbidity.

The current study also underscores the importance of baseline (pre-surgical) PT evaluations and ongoing PT evaluations in order to improve our understanding of the impact of radiation and surgery on shoulder morbidity. Fewer than 50% of patients underwent a pre-surgical PT assessment, and only 25% of patients underwent PT evaluation prior to surgery, prior to radiation, and post-radiation. In addition, >10% of patients did not undergo any PT evaluation. Therefore, we are likely not accurately capturing shoulder/arm morbidity rates and without pre-surgical baseline assessments, we would not be able to determine if an identified morbidity is related to treatment. Similar to the lymphedema screening program at the Massachusetts General Hospital that stresses the importance of baseline measurements and ongoing evaluations for lymphedema (29, 30), the same principles should apply for the screening, early detection, and treatment of shoulder morbidity.

Due to the retrospective nature, our study has several limitations. First, only 20% of patients had a q-DASH performed and not all of these patients had a q-DASH prior to surgery. In addition, the small sample size of patients that completed the q-DASH may have limited the power to detect a significant difference between IMRT and 3DCRT patients, though the trend in favor of IMRT was strong. Due to the retrospective nature, quantitative assessments of shoulder function were not routinely performed in all patients. While we demonstrated that IMRT use resulted in less absolute and relative volume of shoulder OAR exposed to 20–50 Gy, it is possible that changes to the pectoralis muscles from surgery and radiation have a more significant impact on shoulder and arm function such that IMRT use would not necessarily result in a clinically meaningful reduction in shoulder morbidity over time. In addition, radiation dose to the latissimus dorsi (and other structures predominantly located in the back OAR) may have significantly impact shoulder/arm morbidity, and IMRT use has a more modest improvement in reduction of volume of back OAR receiving moderate/high-dose radiation compared to 3DCRT. Last, while it is hypothesized that the moderate and especially high doses (V40–V50 Gy) have a more significant impact on shoulder/arm morbidity, it is possible that the excessive volume of shoulder OAR and back OAR exposed to 5–10 Gy with IMRT may contribute to long-term shoulder/arm morbidity.

In summary, we have demonstrated that compared to 3DCRT, IMRT use results in a significant reduction in the relative and absolute volume of shoulder receiving 20–50 Gy in breast cancer patients receiving RNI. In the subset of 82 patients that completed a q-DASH>6 months from the end of RNI, 94% of which were experiencing arm/shoulder symptoms, patients with IMRT had numerically lower scores, indicating less severe shoulder morbidity. This hypothesis-generating study suggests that the IMRT technique may be an intervention to help reduce long-term shoulder morbidity in patients receiving RNI. These data support prospective evaluation of shoulder/arm function after RNI by radiation treatment technique using both patient-reported outcomes and quantitative functional assessments, which is currently underway (31).

## Data Availability Statement

The raw data supporting the conclusions of this article will be made available by the authors, without undue reservation.

## Ethics Statement

The studies involving human participants were reviewed and approved by Ohio State University Internal Review Board. Written informed consent for participation was not required for this study in accordance with the national legislation and the institutional requirements.

## Author Contributions

JB was involved in the conception and design, data collection, analysis, and writing. JW was involved in the conception and design and writing. DD was involved in the data collection and writing. All authors contributed to the article and approved the submitted version.

## Conflict of Interest

The authors declare that the research was conducted in the absence of any commercial or financial relationships that could be construed as a potential conflict of interest.

## References

[1] McGale P Taylor C Correa C Cutter D Duane F Ewertz M. Effect of radiotherapy after mastectomy and axillary surgery on 10-year recurrence and 20-year breast cancer mortality: meta-analysis of individual patient data for 8135 women in 22 randomised trials. Lancet (2014) 383:2127–35. 10.1016/S0140-6736(14)60488-8 PMC5015598 24656685

[2] Poortmans PM Collette S Kirkove C Van Limbergen E Budach V Struikmans H. Internal Mammary and Medial Supraclavicular Irradiation in Breast Cancer. N Engl J Med (2015) 373:317–27. 10.1056/NEJMoa1415369 26200978

[3] Whelan TJ Olivotto IA Parulekar WR Ackerman I Chua BH Nabid A. Regional Nodal Irradiation in Early-Stage Breast Cancer. N Engl J Med (2015) 373:307–16. 10.1056/NEJMoa1415340 PMC4556358 26200977

[4] Blomqvist L Stark B Engler N Malm M. Evaluation of arm and shoulder mobility and strength after modified radical mastectomy and radiotherapy. Acta Oncol (2004) 43:280–3. 10.1080/02841860410026170 15244252

[5] Harrington S Padua D Battaglini C Michener LA Giuliani C Myers J. Comparison of shoulder flexibility, strength, and function between breast cancer survivors and healthy participants. J Cancer Surviv Res Pract (2011) 5:167–74. 10.1007/s11764-010-0168-0 21225372

[6] Hidding JT Beurskens CH van der Wees PJ van Laarhoven HW Nijhuis-van der Sanden MW. Treatment related impairments in arm and shoulder in patients with breast cancer: a systematic review. PloS One (2014) 9:e96748. 10.1371/journal.pone.0096748 24816774 PMC4016041

[7] Hojris I Andersen J Overgaard M Overgaard J. Late treatment-related morbidity in breast cancer patients randomized to postmastectomy radiotherapy and systemic treatment versus systemic treatment alone. Acta Oncol (2000) 39:355–72. 10.1080/028418600750013131 10987233

[8] Johansen J Overgaard J Blichert-Toft M Overgaard M. Treatment morbidity associated with the management of the axilla in breast-conserving therapy. Acta Oncol (2000) 39:349–54. 10.1080/028418600750013122 10987232

[9] Kwan W Jackson J Weir LM Dingee C McGregor G Olivotto IA. Chronic arm morbidity after curative breast cancer treatment: Prevalence and impact on quality of life. J Clin Oncol (2002) 20:4242–8. 10.1200/JCO.2002.09.018 12377968

[10] Lee TS Kilbreath SL Refshauge KM Herbert RD Beith JM. Prognosis of the upper limb following surgery and radiation for breast cancer. Breast Cancer Res Treat (2008) 110:19–37. 10.1007/s10549-007-9710-9 17899373

[11] Levangie PK Drouin J. Magnitude of late effects of breast cancer treatments on shoulder function: a systematic review. Breast Cancer Res Treat (2009) 116:1–15. 10.1007/s10549-008-0246-4 19031114

[12] Nesvold IL Dahl AA Lokkevik E Mengshoel AM Fossa SD. Arm and shoulder morbidity in breast cancer patients after breast-conserving therapy versus mastectomy. Acta Oncol (2008) 47:835–42. 10.1080/02841860801961257 18568481

[13] Donker M van Tienhoven G Straver ME Meijnen P van de Velde CJ Mansel RE. Radiotherapy or surgery of the axilla after a positive sentinel node in breast cancer (EORTC 10981-22023 AMAROS): a randomised, multicentre, open-label, phase 3 non-inferiority trial. Lancet Oncol (2014) 15:1303–10. 10.1016/S1470-2045(14)70460-7 PMC4291166 25439688

[14] Lipps DB Sachdev S Strauss JB. Quantifying radiation dose delivered to individual shoulder muscles during breast radiotherapy. Radiother Oncol (2017) 122:431–6. 10.1016/j.radonc.2016.12.032 PMC5366257 28129897

[15] Ho AY Ballangrud A Li G Gupta GP McCormick B Gewanter R. Long-Term Pulmonary Outcomes of a Feasibility Study of Inverse-Planned, Multibeam Intensity Modulated Radiation Therapy in Node-Positive Breast Cancer Patients Receiving Regional Nodal Irradiation. Int J Radiat Oncol Biol Phys (2019) 103:1100–8. 10.1016/j.ijrobp.2018.11.045 PMC6777954 30508620

[16] Jagsi R Griffith KA Moran JM Ficaro E Marsh R Dess RT. A Randomized Comparison of Radiation Therapy Techniques in the Management of Node-Positive Breast Cancer: Primary Outcomes Analysis. Int J Radiat Oncol Biol Phys (2018) 101:1149–58. 10.1016/j.ijrobp.2018.04.075 PMC6938718 30012527

[17] Harrington S Michener LA Kendig T Miale S George SZ. Patient-reported upper extremity outcome measures used in breast cancer survivors: a systematic review. Arch Phys Med Rehabil (2014) 95:153–62. 10.1016/j.apmr.2013.07.022 PMC4162515 23932969

[18] Kennedy CA Beaton DE Smith P Van Eerd D Tang K Inrig T. Measurement properties of the QuickDASH (disabilities of the arm, shoulder and hand) outcome measure and cross-cultural adaptations of the QuickDASH: a systematic review. Qual Life Res (2013) 22:2509–47. 10.1007/s11136-013-0362-4 23479209

[19] Kennedy CA BD Solway S McConnell S Bombardier C. Disabilities of the Arm, Shoulder and Hand (DASH). The DASH and QuickDASH Outcome Measure User’s Manual. 3rd ed. Toronto: Institute for Work & Health (2011).

[20] Bazan J DiCostanzo D Kuhn K Majithia L Quick A Gupta N. Likelihood of unacceptable normal tissue doses in breast cancer patients undergoing regional nodal irradiation in routine clinical practice. Pract Radiat Oncol (2017) 7:154–60. 10.1016/j.prro.2016.10.012 28094211

[21] Bazan JG Healy E Beyer S Kuhn K DiCostanzo D Smith TL. Clinical Effectiveness of an Adaptive Treatment Planning Algorithm for Intensity Modulated Radiation Therapy Versus 3D Conformal Radiation Therapy for Node-Positive Breast Cancer Patients Undergoing Regional Nodal Irradiation/Postmastectomy Radiation Therapy. Int J Radiat Oncol Biol Phys (2020) 108(5):1159–71. 10.1016/j.ijrobp.2020.07.027 32711036

[22] NCT01872975. Standard or comprehensive radiation therapy in treating patients with early-stage breast cancer previously treated with chemotherapy and surgery. (2020). Available at: https://clinicaltrials.gov/ct2/show/NCT01872975.

[23] White J Tai A Arthur D Buchholz T MaDonald S Marks L. Breast cancer atlas for radiation therapy planning: consenus definitions. (2011).

[24] Johansen S Fossa K Nesvold IL Malinen E Fossa SD. Arm and shoulder morbidity following surgery and radiotherapy for breast cancer. Acta Oncol (2014) 53:521–9. 10.3109/0284186X.2014.880512 24495044

[25] The Quick DASH Outcome Measure: Information for Users. Available at: https://dash.iwh.on.ca/sites/dash/files/downloads/quickdash_info_2010.pdf.

[26] Lipps DB Leonardis JM Dess RT McGinnis GJ Marsh RB Strauss JB. Mechanical properties of the shoulder and pectoralis major in breast cancer patients undergoing breast-conserving surgery with axillary surgery and radiotherapy. Sci Rep (2019) 9:17737. 10.1038/s41598-019-54100-6 31780712 PMC6882786

[27] Haviland JS Mannino M Griffin C Porta N Sydenham M Bliss JM. Late normal tissue effects in the arm and shoulder following lymphatic radiotherapy: Results from the UK START (Standardisation of Breast Radiotherapy) trials. Radiother Oncol (2018) 126:155–62. 10.1016/j.radonc.2017.10.033 PMC5807031 29153463

[28] Crosbie J Kilbreath SL Dylke E Refshauge KM Nicholson LL Beith JM. Effects of mastectomy on shoulder and spinal kinematics during bilateral upper-limb movement. Phys Ther (2010) 90:679–92. 10.2522/ptj.20090104 20223945

[29] Brunelle C Skolny M Ferguson C Swaroop M O’Toole J Taghian AG. Establishing and sustaining a prospective screening program for breast cancer-related lymphedema at the massachusetts general hospital: lessons learned. J Pers Med (2015) 5:153–64. 10.3390/jpm5020153 PMC4493493 26011383

[30] Gillespie TC Sayegh HE Brunelle CL Daniell KM Taghian AG. Breast cancer-related lymphedema: risk factors, precautionary measures, and treatments. Gland Surg (2018) 7:379–403. 10.21037/gs.2017.11.04 30175055 PMC6107585

[31] NCT03786354. Intensity Modulated Radiation Therapy or 3-Dimensional Conformal Radiation Therapy in Treating Patients With Lymph-Node Positive Breast Cancer. Available at: https://clinicaltrials.gov/ct2/show/NCT03786354

